# A cross-sectional survey of poultry management systems, practices and antimicrobial use in relation to disease outbreak in Pakistan

**DOI:** 10.1186/s13104-025-07220-4

**Published:** 2025-04-08

**Authors:** Farrukh Saleem, Aqsa Ameer, Farhan Afzal, Muhammad Usman, Hamid Irshad, Sadia Sattar, Umer Zeeshan Ijaz, Sundus Javed

**Affiliations:** 1 Department of Biosciences, COMSATS University Islamabad, Islamabad, Pakistan; 2 National Veterinary Laboratories, Ministry of National Food Security and Research, Islamabad, Pakistan; 3 Poultry Research Institute Punjab, Rawalpindi, Pakistan; 4 Animal Sciences Institute, National Agricultural Research Center, Islamabad, Pakistan; 5 Water & Environment Research Group, Mazumdar-Shaw Advanced Research Centre, University of Glasgow, Glasgow, UK; 6 Department of Molecular and Clinical Cancer Medicine, University of Liverpool, Liverpool, UK; 7 College of Science and Engineering, University of Galway, Galway, Ireland

**Keywords:** Survey, Management practices, Disease outbreak, Antimicrobials, Zoonosis

## Abstract

**Objectives:**

The study aimed to examine how management practices, farming setup and breed influence disease outbreaks. It also sought to investigate the frequency and types of antimicrobials used, as well as the relationship between antimicrobial usage and disease occurrences.

**Methods:**

We conducted a survey of 140 poultry farms [Broiler farms = 66; Layer farms = 36; Local (Desi and its crosses) farms = 38] across major poultry producing regions of Pakistan. The gathered information covered demographics as well as the farming associated parameters including size, type of the farms, management practices, breeds raised, disease outbreak and antimicrobials use.

**Results:**

Using contingency analyses and log binomial regression models, we identified Broiler control sheds at high risk of disease. Diseases such as Avian Influenza, Newcastle Disease, and Fowl Typhoid were frequently reported and their outbreaks were associated with low cleaning frequency, high stocking density, bedding material using rice husk, and canola as a major feed ingredient. Farmer education was associated with a decrease in disease outbreak. Antimicrobial use was associated with farming experience, farm size, type and breed.

**Conclusion:**

High disease incidence is associated with management practices and breed types across various farm setups. Experienced Broiler farmers often report disease outbreaks and use antimicrobials more frequently. Educated farmers, however, experience fewer outbreaks and can better regulate antimicrobial usage.

**Supplementary Information:**

The online version contains supplementary material available at 10.1186/s13104-025-07220-4.

## Introduction

Poultry industry is an important sector of Pakistani agriculture and plays a central role in country’s economic development, bringing in around 1.5 million jobs annually. Currently, there are around 15,000 to 20,000 farms, producing > 2,250-million-kilogram meat and 18,000 million table eggs annually [1], with an annual growth of 7.3% [2]. The industry expanded from 1960 to 1980s, but it was not without its perils. Huge production losses were reported due to outbreaks of different infections such as avian influenza, Newcastle disease, fowl typhoid, Marek’s disease, etc. [3–6]. Circumventing these crises prompted a shift in poultry sector towards antimicrobials (AMs) usage as therapeutics and growth promoters [7–9]. In the past decade, antimicrobial resistance (AMR) has posed a major threat to global health due to higher rates of mortality and illnesses in humans and animals [10]. It has been reported that unmediated transmission of AMR bacteria occurs from livestock, especially poultry, to handlers and vice versa [11–13]. The general public is the predominant recipient of AMR bacteria involving various factors [14, 15]. Although farmers are concerned about the health of birds, they are unaware of the risk factors associated with disease outbreaks, transmission of zoonotic infections and AMR [16, 17]. Hence, understanding of farmer perspective and management practices is necessary such as following hygienic measures may enable antimicrobials and disease free rearing of poultry [18, 19].

Using an interview-based survey, the present study was designed to establish baseline information regarding different farming systems, management practices, training and awareness of farmers regarding outbreak, farmer health and antimicrobials use.

## Materials and methods

### Expert consultation, study design and categorization of farms

**Fig. 1:**
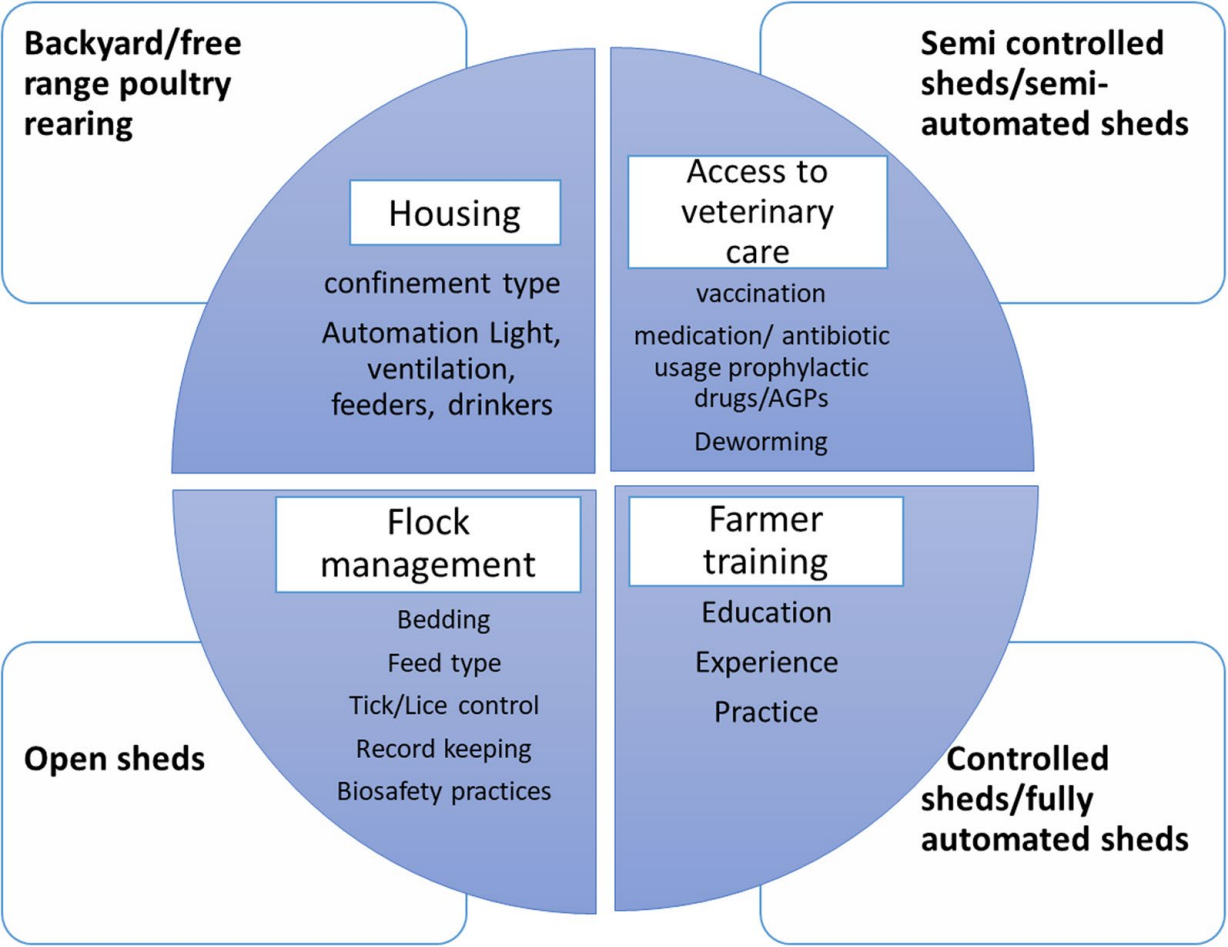
Comparison of main management parameters in terms of zoonotic transmission risk in different poultry farming setups

A cross-sectional field survey was conducted, with a non-experimental research design where researchers recorded variables and tested their effects on disease outbreak information and antimicrobials usage using statistical methods. Prior to formulation of questionnaire, a panel of 15 poultry experts [veterinarians *(n* = *5);* veterinary pathologists *(n* = *3);* veterinary pharmacists *(n* = *2)* and farmers *(n* = *5)*] were consulted. Focus group discussions lead to selection of poultry farms and to identify major poultry farming setups according to management practices, based on their field knowledge and experience (Fig. 1). Farms were limited to those that were easily accessible, and were registered with the Poultry Research Institute (PRI) Punjab to ensure availability of verifiable intermittent disease outbreak data, and where the farms had a historical legacy of routinely submitting their samples to PRI for diagnoses of disease. Furthermore, focus group discussions also led to the final version of questionnaire which was first filled by the experts and were considered as quality control.

The study was conducted in the major poultry producing regions of Punjab and Khyber Pakhtunkhwa including Islamabad capital territory in Pakistan, during February—November 2022. Data was gathered through a questionnaire based on one-on-one interviews of 140 poultry farmers. Majority of the farms included in the survey were individual holdings and not belonging to the corporate sector. The participant farmers were briefed about the broad purpose of the questionnaires *i.e.,* general farm management practices.

### Questionnaire design and data collection

**Fig. 2:**
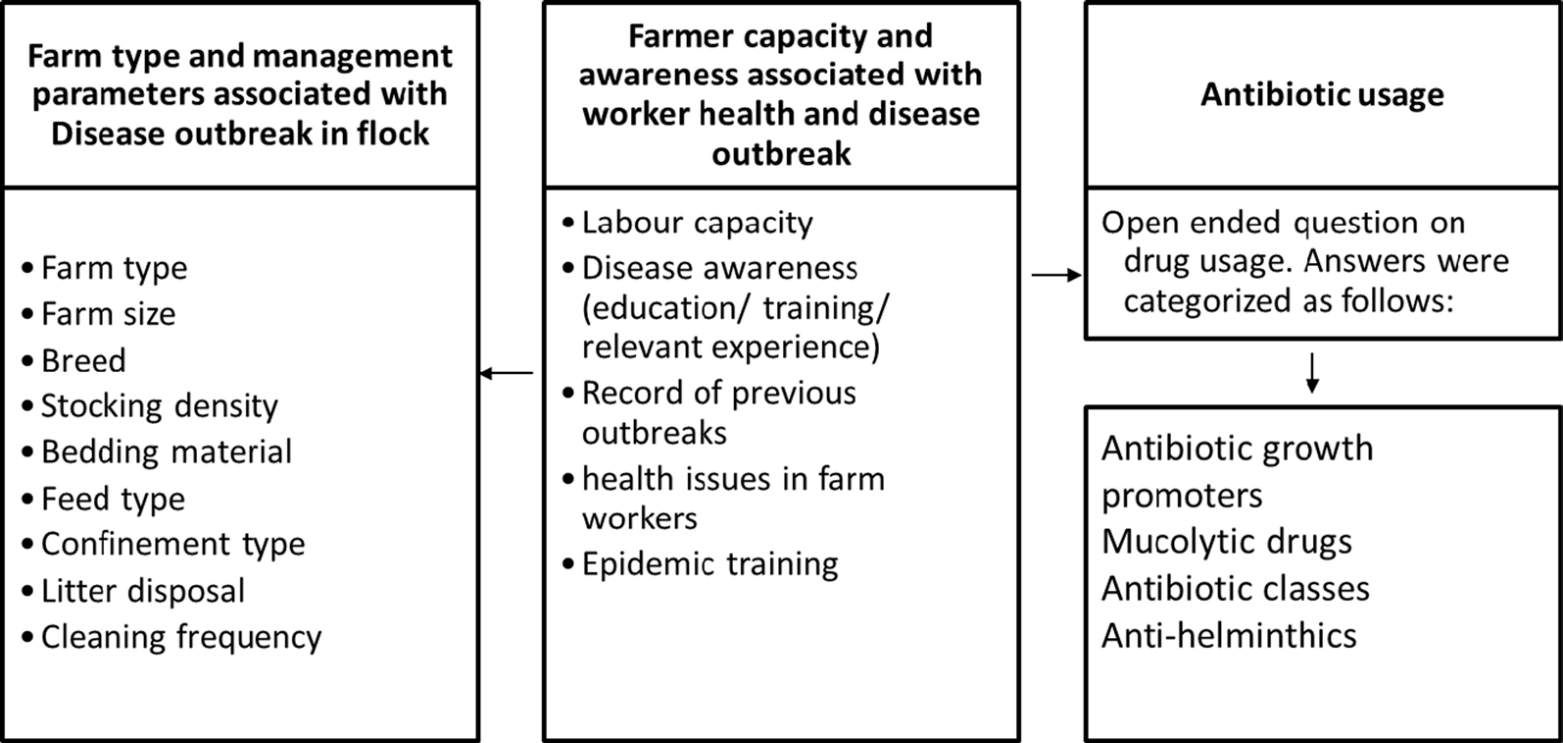
Framework for recording major parameters associated with antimicrobial use and disease outbreak

Based on expert consultation and literature survey, a broad-range questionnaire was drafted including ~ 25 closed and open-ended questions (S5 Fig). The conceptual framework for recording major parameters associated with antimicrobials use and disease outbreak is summarized in Fig. 2. An outbreak is previously defined by the death of two birds (at the minimum) of the same species with similar clinical signs in corresponding farms in the same month, one or two months prior. Here, we have followed a strict criterion for considering a disease as an outbreak. The respondents were specifically asked about their past experiences with high mortality (> 50% of the flock) and if they know that the disease was also reported in nearby farms. However, the latter criterion could not be strictly fulfilled either due to entry restrictions, reluctant behavior or closures due to COVID-19 pandemic. The in-person interviews with farmers were conducted with the questionnaire administered by a trained veterinarian. The questions were explained in the local language and the interviews were conducted on site to ensure reliability of the data collected. For descriptive questions, particularly on antibiotic usage, the veterinarian synthesized the information into different categories.

### Statistical analysis

For the categorical data, to see if any two covariates have a relationship, we constructed a contingency table and used ${\chi }^{2}$ test of independence using chisq.test() function in R [20]. Based on http://www.sthda.com/english/wiki/chi-square-test-of-independence-in-r, and where the relationship existed, we then calculated ${\chi }^{2}$ residuals for individual rows and columns of the contingency table. These were drawn using R’s corrplot [21] package where positive values in cells specify an attraction (positive association; blue) between the corresponding row and column variables whilst negative values implies a repulsion (negative association; red) between the corresponding row and column variables. To get the relative risks for disease outbreak, we have used generalized linear models (GLMs) with log link functions to binomial data using R’s logbin package [22]. To generate the regression tables, we have used tab_model() function from R’s sjPlot package [23] which also facilitated confidence interval display. In some cases, where we had more than two categories in the outcome variable, we have used multinomial logistic regression using multinom() function from R’s nnet package [24] with recommendations given in https://stats.oarc.ucla.edu/r/dae/multinomial-logistic-regression/. For UpSet plots, ggupset repository was used: https://github.com/const-ae/ggupset.

## Results

### Respondent characteristics and scope of survey

**Fig. 3:**
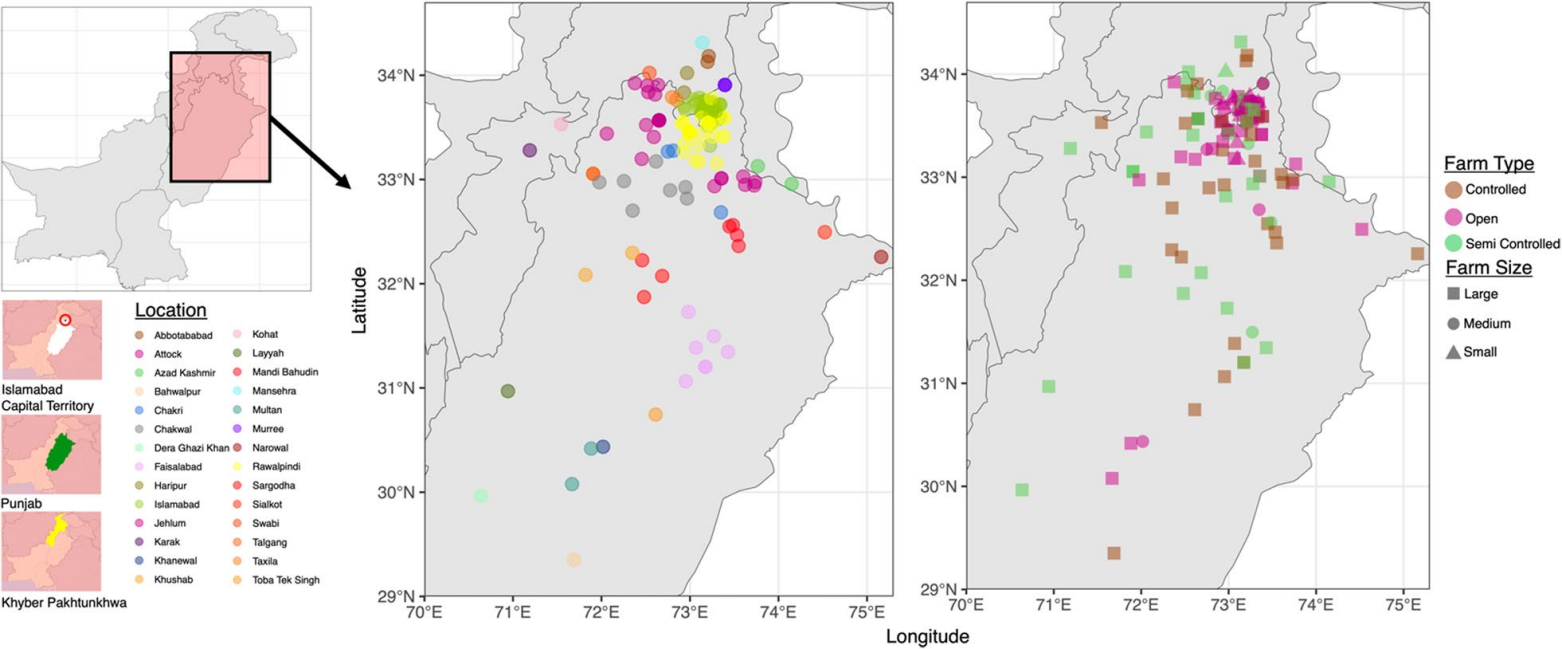
Location, size, and types of farms included in survey, with majority of them belonging to Punjab region, with some located in Khyber Pakhtunkhwa. Note that these are the major poultry producing regions. Maps created in R using sf package [65] and R’s rnaturalearth package [66]

**Fig. 4:**
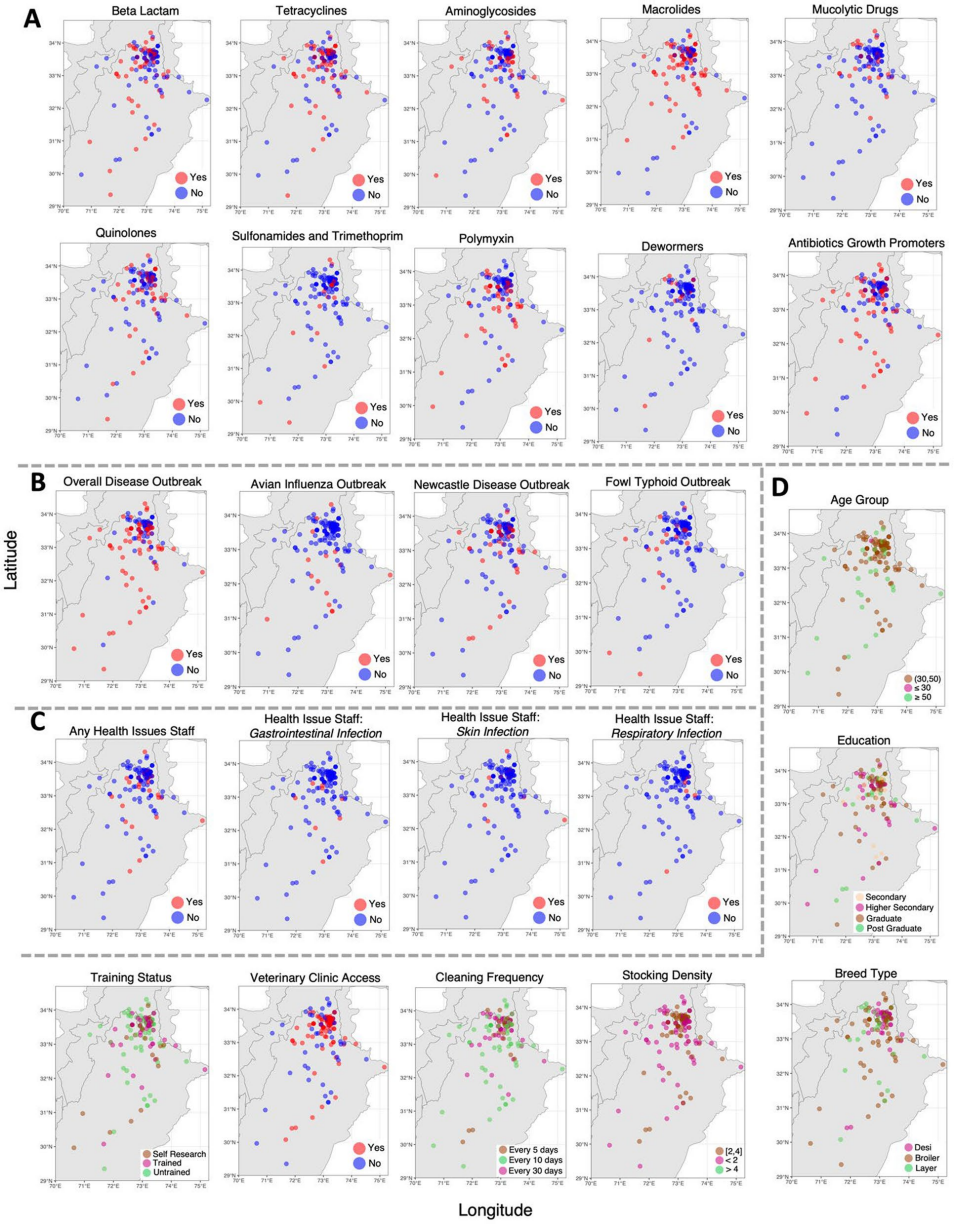
Distribution of key parameters associated with the farms, categorized under (**A**) veterinary drugs, **B** disease outbreak in birds, **C** health issues reported in farmer workers, and **D** other parameters including farm management. Maps created in R using sf package [65] and R’s rnaturalearth package [66]

Based on contingency analysis, we observed significant relationships between various parameters and incidence of disease outbreak (Fig S1). Log binomial regression statistics was used to calculate the prevalence ratios of disease outbreak. Farmers with minimal formal education are more inclined towards farming hybrid breeds and face more health issues in their flocks. 82.86% farmers with higher secondary qualification reported different disease outbreaks including fowl typhoid, avian influenza and Newcastle disease. In contrast, farmers with high education level i.e., graduation and post-graduation, reported less outbreaks (25.81% prevalence with 64% reduction in risk of disease outbreak as compared to those with secondary education) and preferred raising Local (Desi and its crosses) breeds. Furthermore, experienced farmers reported health issues in birds with a large number involved in rearing Broiler birds in intensive setups. No significant association of farmer’s training status with disease outbreaks was observed (Table 2; Figs. 3, 4).

### Different types of commercial poultry farming setups and disease spread

Major poultry setups, along with backyard farming were identified by the poultry experts, and were based on typical management practices followed locally (Table 1).

**Table 1:** Baseline difference between different poultry farming setups depending on the management practices

Management parameters	Backyard/free range poultry rearing	Open sheds	Semi controlled sheds/semi-automated sheds	Controlled sheds/fully automated sheds
Housing	No proper sheds	Pillars fixed with mesh and small protection wall	Fixed solid walls	Fixed solid walls
Ventilation	Natural	Natural	Manual exhaust fans	Automated ventilation system
Lightening	Sunlight	Sunlight/electric bulbs	Electric bulbs	Electric bulbs
Roaming area	No boundaries	Shed area	Shed area	Shed area
Stocking density	NA	Broiler: 1.5–2 ft^2^/bird Layer: 2.5–4 ft^2^/bird	Broiler: 1–1.5 ft^2^/bird Layer: 2–3 ft^2^/bird	Broiler: 0.75–1 ft^2^/bird Layer: 1.5–2 ft^2^/bird
Drinkers	Manual	Manual	Manual/automated	Automated
Feeders	Manual	Manual	Manual	Automated
Feed type	Kitchen waste, fodder, vegetables, insects	Commercial feed with additives and growth promoters	Commercial feed with additives and growth promoters	Commercial feed with additives and growth promoters
Vaccination	No vaccination	Scheduled vaccination	Scheduled vaccination	Scheduled vaccination
Medication	No medication	Prophylactics and treatment	Prophylactics and treatment	Prophylactics and treatment
Deworming	Not followed	Followed	Followed	Followed
Tick/Lice Control	No control	Controlled	Controlled	Controlled
Workers	No staff	Supervisor and care takers	Veterinarian, supervisor and care takers	Veterinarian, supervisor, electrician, plumber and care takers
Biosecurity	Not implemented	Not implemented	Implemented	Implemented
Record keeping	No	Yes	Yes	Yes

Commercial poultry farming setups including open (44.3%), semi-controlled (21.4%) and controlled sheds (34.3%) were targeted. Disease outbreak was observed in 93.33% of controlled sheds which are predominantly involved in Broiler rearing as compared to the open sheds (60% reduced risk of disease outbreak as compared to controlled sheds) or semi controlled (44% reduced risk of disease outbreak as compared to controlled sheds) setups (Table 2).

**Table 2:** Major risk predictors associated with disease outbreaks at poultry farms by fitting different regression models represented by M numbers

Predictor		*β-* coefficients	Count (n = 140)	Disease outbreak reported, n = 76 (54.29%)	Prevalence Ratio (95% CI)	Significance
M1	Intercept		NA		0.72 (0.54—0.96)	*
	Farmer’s Education	Secondary	18	13 (72.22%)	REF	NA
		Higher secondary	35	29 (82.86%)	1.15 (0.83—1.59)	NS
		*Graduation*	*56*	*26 (46.43%)*	**0.64** (0.43—0.96)	*
		*Post-graduation*	*31*	*8 (25.81%)*	**0.36** (0.18—0.69)	**
M2	Intercept		NA		0.27 (0.15—0.48)	***
	Farming Experience	Less than 5 years	30	8 (26.67%)	REF	NA
		5 to 10 years	65	27 (41.54%)	1.56 (0.81—3.01)	NS
		***More than 10 years***	***45***	***41 (91.11%)***	**3.42** (1.87—6.23)	***
M3	Intercept		NA		0.55 (0.44 —0.68)	***
	Epidemic Training Status	Untrained	66	36 (54.55%)	REF	NA
		Self-research	23	14 (60.87%)	1.12 (0.75—1.66)	NS
		Trained	51	26 (50.98%)	0.93 (0.66—1.32)	NS
M4	Intercept		NA		0.93	NS
	Farm Type	Controlled	30	28 (93.33%)	REF	NA
		*Semi controlled*	*48*	*25 (52.08%)*	**0.56** (0.42—0.74)	***
		*Open*	*62*	*23 (30.09%)*	**0.40** (0.28—0.56)	***
M5	Intercept		NA		0.36 (0.25—0.52)	***
	Labor Capacity	Less than 3	50	18 (36.00%)	REF	NA
		3 to 5	57	27 (47.37%)	1.32 (0.83—2.08)	NS
		***More than5***	***33***	***31 (93.93%)***	**2.61** (1.79—3.81)	***
M6	Intercept		NA		0.73 (0.63—0.84)	***
	Breed Type	Broiler	66	48 (72.73%)	REF	NA
		*Layer*	*36*	*15 (41.67%)*	**0.57** (0.38—0.87)	**
		*Desi and crosses*	*38*	*13 (34.21%)*	**0.47** (0.30—0.75)	**
M7	Intercept		NA		0.33 (0.13—0.84)	*
	Confinement Type	No confinement	9	3 (33.33%)	REF	NA
		Open house with mesh	58	22 (37.93%)	1.14 (0.43—3.03)	NS
		Closed house with solid walls	73	51 (69.86%)	2.10 (0.82—5.34)	NS
M8	Intercept		NA		0.68 (0.58—0.79)	***
	Flock Management	All-in-all-out	74	50 (67.57%)	REF	NA
		*Multiple flocks and all-in-all-out*	*55*	*21 (38.18%)*	**0.57** (0.39—0.82)	**
		Continuous topping	11	5 (45.45%)	0.67 (0.35—1.31)	NS
M9	Intercept	Intercept	NA		0.36 (0.21—0.61)	***
	Cleaning Frequency	Every 5 days	25	9 (36.00%)	REF	NA
		Every 10 days	48	20 (41.67%)	1.16 (0.62—2.15)	NS
		***Every 30 days***	***67***	***47 (70.15%)***	**1.95** (1.13—3.36)	*
M10	Intercept		NA		0.67 (0.38—1.17)	NS
	Litter Disposal	Open place	6	4 (66.67%)	REF	NA
		Drain	36	23 (68.89%)	0.96 (0.52—1.78)	NS
		Pit	98	49 (50.00%)	0.75 (0.41—1.37)	NS
M11	Intercept		NA		0.72 (0.63—0.83)	***
	Stocking Density	Less than 2 ft^2^/bird	72	52 (72.22%)	REF	NA
		*2 to 4 ft*^*2*^*/bird*	*66*	*23 (38.85%)*	**0.48** (0.34—0.69)	***
		More than 4 ft^2^/bird	2	1 (50.00%)	0.69 (0.17—2.79)	NS
M12	Intercept		NA		0.36 (0.26—0.48)	***
	Major Feed Ingredient: Canola	No	73	26 (35.62%)	REF	NA
		***Yes***	***67***	***50 (72.63%)***	**2.10** (1.49—2.94)	***
M13	Intercept		NA		0.67 (0.55—0.82)	***
	Major Feed Ingredient: Wheat	No	46	31 (67.39%)	REF	NA
		*Yes*	*94*	*45 (47.87%)*	**0.71** (0.53—0.95)	*
M14	Intercept		NA		0.54 (0.42—0.69)	***
	Veterinary Clinic Access	No	52	28 (53.85%)	REF	NA
		Yes	88	48 (54.55%)	1.01 (0.74—1.39)	NS
M15	Intercept		NA		0.43 (0.35—0.53)	***
	Health Issue Staff	No	109	47 (43.12%)	REF	NA
		***Yes***	***31***	***29 (93.55%)***	**2.17** (1.72—2.74)	***

The significant predictors that cause an increase in disease outbreak are shown with a bold italic font, whilst those that cause a decrease in outbreak are shown with an Italic font as compared to reference (REF), with the prevalence ratio/risk ratio shown in bold

^*^NA refers to Not Applicable; NS refers to Non-significant

^*^p < 0.05 **p < 0.01 ***p < 0.001

Furthermore, we categorized the farms as small, medium and large, with maximum disease outbreak reported in large farms. These large farms have greater labor capacity and therefore pose a higher risk of disease spread (93.93% of the farms with labor capacity of more than 5 laborers). Majority of the farms included in the study were raising Broiler (47%), in controlled sheds and are a popular choice due to quick turnover times (Table 2; Fig S2-S3).

### Frequency of common disease outbreaks reported with major risk predictors

Majority of the farmers reported Newcastle disease, avian influenza and fowl typhoid outbreaks (Fig. 4B). Farmers also reported few other infections such as Marek’s disease, bronchitis, coccidiosis, colibacillosis but they were not sure about the exact mortality and spread so we did not consider them as an outbreak. Out of a total 140 poultry farms, avian influenza (AI), Newcastle disease (ND) and fowl typhoid (FT) outbreaks were reported in 20 (14.29%), 38 (27.14%) and 18 (12.86%) farms, respectively, and found disease risk predictors including education, training status, farm type, breed type, cleaning frequency, litter disposal method and feed ingredients and calculated prevalence ratios. Farming experience of over 10 years was associated with a 3.42 folds increase in outbreak reporting (Table 2). It must be noted that the questions asked during the interviews did not indicate any time frame for disease reporting and with greater farming experience, increased chances of encountering a disease outbreak become likely. Further, 32.26% of farms with AI outbreak, 45.16% with ND, and 19.36% with FT also reported health issues in the farm workers (S1-S3 Tables).

Compared to controlled sheds, semi controlled and open sheds reported fewer disease outbreaks. As for the breed types, raising Local breeds and Layer resulted in 43% and 53% reduced risk in disease outbreaks, respectively, compared to raising Broiler (Fig S2-S3). Certain parameters associated with farm management also correlated with risk of disease outbreak e.g., labor capacity of over 5 workers compared to < 3 workers was associated with 2.61 fold increase in outbreak risk. Decreasing farm cleaning frequency from every 5 days to every 30 days increased outbreak risk 1.95 times. 72.22% of the farms with stocking density of < 2 ft^2^/bird, reported disease outbreak. In contrast, decreasing stocking density from < 2ft^2^ to 2–4 ft^2^, decreases the risk of disease outbreak by 48%. Canola as a feed ingredient, increased the disease risk 2.10 folds and wheat decreased the risk of disease outbreak by 29% (Fig S4). In contrast to wood shaving, rice husk (bedding material) was associated with high disease risk (Table 2; Fig S2-S3).

### Zoonosis emergence with reference to farmer training and management practices

Farmers who reported diseases in their birds also reported concomitant health issues in their staff (increased risk of disease outbreak by 12.21 times). 38.16% of the farms with disease outbreak history coincided with different human infections (Fig. 4C). AI and ND outbreaks were associated with 2.86- and 2.05-folds increase in risk of health issues amongst farm workers, respectively (S1-S2 Tables). The majority of larger farms, with controlled setups and higher labor capacity, are found to be at a higher risk of zoonosis. As compared to controlled sheds, we observed 58% and 56% reduced risk of health issues in farmers associated with semi-controlled and open sheds, respectively. A decreased risk for disease amongst workers was seen with farmers raising Local breeds. (0.37 or 63% reduction in risk as compared to raising Broiler). We did not ask the farmers about any specific disease occurrence, but based on the information gathered, we broadly categorized the infections as respiratory, digestive and skin-related infections (Table 3).

**Table 3:** Major risk predictors associated with health issues in poultry farmers by fitting different regression models represented by M numbers

Predictor		*β-* coefficients	Count (n = 140)	Health issues staff reported, n = 31 (22.14%)	Prevalence Ratio (95% CI)	Significance
M1	Intercept		NA		0.22 (0.09—0.53)	***
	Farmer’s Education	Secondary	18	4 (22.22%)	REF	NA
		Higher secondary	35	11 (31.43%)	1.41 (0.52—3.82)	NS
		Graduation	56	10 (17.86%)	0.80 (0.29—2.25)	NS
		Post-graduation	31	6 (19.35%)	0.87 (0.28—2.68)	NS
M2	Intercept		NA		0.07 (0.02—0.25)	***
	Farming Experience	Less than 5 years	30	2 (6.67%)	REF	NA
		5 to 10 years	65	14 (21.54%)	3.23 (0.78—13.33)	NS
		***More than 10 years***	***45***	***15 (33.33%)***	**5.00** (1.23—20.30)	*
M3	Intercept		NA		0.23 (0.15—0.35)	***
	Epidemic Training Status	Untrained	66	15 (22.73%)	REF	NA
		Self-research	23	4 (17.39%)	0.77 (0.28—2.07)	NS
		Trained	51	12 (23.53%)	1.04 (0.53—2.01)	NS
M4	Intercept		NA		0.40	***
	Farm Type	Controlled	30	12 (40.00%)	REF	NA
		*Semi controlled*	*48*	*8 (16.67%)*	**0.42** (0.19—0.90)	*
		*Open*	*62*	*11 (17.74%)*	**0.44** (0.22—0.89)	*
M5	Intercept		NA		0.12 (0.06—0.25)	***
	Labor Capacity	Less than 3	50	6 (12.00%)	REF	NA
		3 to 5	57	11 (19.29%)	1.61 (0.64—4.03)	NS
		***More than5***	***33***	***14 (42.42%)***	**3.54** (1.51—8.27)	**
M6	Intercept		NA		0.29 (0.20—0.42)	***
	Breed Type	Broiler	66	19 (28.79%)	REF	NA
		Layer	36	8 (22.22%)	0.77 (0.38—1.58)	NS
		*Desi and crosses*	*38*	*4 (10.51%)*	**0.37** (0.13—1.00)	*
M7	Intercept		NA		0.27 (0.19—0.39)	***
	Flock Management	All-in-all-out	74	20 (20.03%)	REF	NA
		Multiple flocks and all-in-all-out	55	9 (16.36%)	0.61 (0.30—1.23)	NS
		Continuous topping	11	2 (18.18%)	0.67 (0.18—2.49)	NS
M8	Intercept		NA		0.20 (0.09—0.44)	***
	Cleaning Frequency	Every 5 days	25	5 (20.00%)	REF	NA
		Every 10 days	48	8 (16.67%)	0.83 (0.30—2.28)	NS
		Every 30 days	67	18 (26.87%)	1.34 (0.56—3.23)	NS
M9	Intercept		NA		0.17 (0.03—1.00)	*
	Litter Disposal	Open place	6	1 (16.67%)	REF	NA
		Drain	36	6 (16.67%)	1.00 (0.14—6.91)	NS
		Pit	98	24 (24.49%)	1.47 (0.24—9.09)	NS
M10	Intercept		NA		0.03 (0.01—0.12)	***
	Disease Outbreak	No	64	2 (3.13%)	REF	NA
		***Yes***	***76***	***29 (38.16%)***	**12.21** (3.03—49.21)	***
M11	Intercept		NA		0.18 (0.12—0.26)	***
	Disease Outbreak Avian Influenza	No	120	21 (17.50%)	REF	NA
		***Yes***	***20***	***10 (50.00%)***	**2.86** (1.59—5.13)	***
M12	Intercept		NA		0.20 (0.14—0.29)	***
	Disease Outbreak Fowl Typhoid	No	122	25 (20.49%)	REF	NA
		Yes	18	6 (33.33%)	1.63 (0.78—3.41)	NS

The significant predictors that cause an increase in health issues are shown with a bold italic font, whilst those that cause a decrease in health issues, are shown with an italic font as compared to reference (REF), with the prevalence ratio/risk ratio shown in bold

^*^NA refers to Not Applicable; NS refers to Non-significant

^*^p < 0.05 **p < 0.01 ***p < 0.001

### Antimicrobial usage in different farm setups

Farmers reported disease outbreaks at their farms even when birds are given feed with added antimicrobials as growth promoters or prophylactics (Fig. 4A; S4). Antimicrobials usage was found to be highest amongst medium to large poultry farms following controlled shed system. Concomitant use of macrolides, tetracyclines, beta lactams and quinolones were reported highest in the open sheds (Fig S7A). Macrolides, tetracyclines, beta lactams and quinolones were most frequently employed by 62%, 52%, 47% and 43.5% of farms, respectively. Meanwhile, usage of polymyxin (33.6%), aminoglycosides (26%), and sulfonamides (12.8%), was limited. 45% of farms reported adding antibiotic growth promoters (primarily lincomycin) to bird feeds. 17.8% and 12% farmers also reported using mucolytic and anti-helminthic (dewormers) drugs. Educated farmers reported greater use of macrolides, sulfonamides and anti-helminthics and decreased use of tetracyclines and polymyxin. Experienced farmers reported higher use of beta lactams (4.87 folds high risk), aminoglycosides (7.14 folds high risk) and AGPs (28.34 folds high risk). Medium and large farms were associated with significantly high macrolide, polymyxin and mucolytic drugs use. Large farms also reported high aminoglycoside usage. Semi-controlled and open shed systems had significantly lower aminoglycoside, macrolide, AGPs and anti-helminthic drug use compared to controlled sheds. Farmers rearing Broiler (reference) reported high antimicrobial use and rearing Local and Layer was associated with 0.03 (~ 97% reduction) and 0.188 (~ 81% reduction) AGPs usage, respectively (Table 4).

**Table 4:**
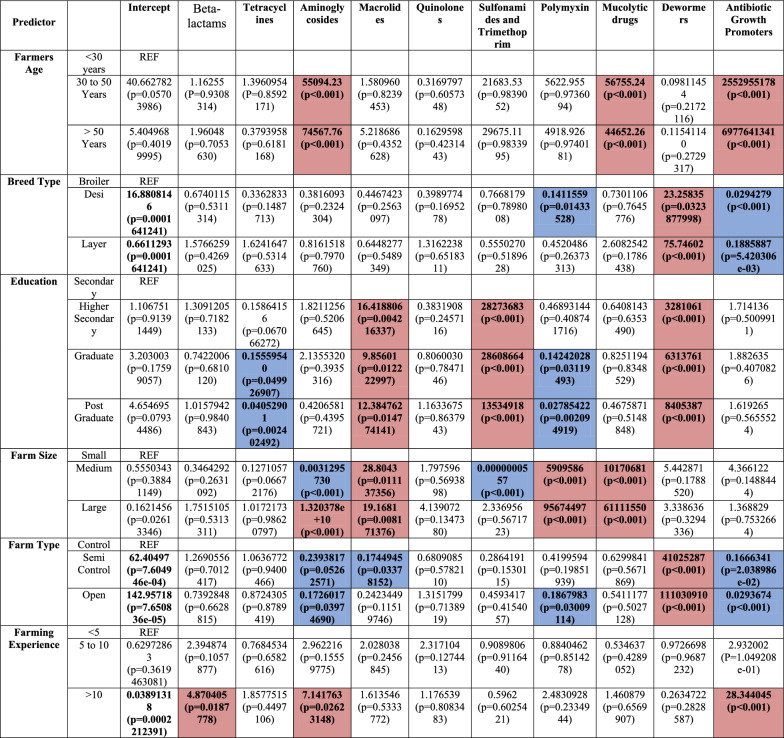
Major risk predictors associated with antimicrobial use when using outcome variables with more than two categories in multinomial logistic regression

The significant predictors that cause an increase in risk as compared to the reference (REF) of using an antimicrobial are shown with red background whilst those that cause decrease in risk as compared to the reference of using the antimicrobials are shown with a blue background

### Identification of the gaps in general farmer’s practices

The farmers response about knowledge on poultry diseases and general practices was highly variable i.e., Newcastle disease (96.4%); avian influenza (84.3%); fowl typhoid (58.57%); infectious bursal disease (52.86%); infectious bronchitis (52.14%); Colibacillosis (42.86%); Marek’s disease (40%); Coccidiosis (37.85%); and Prolapse (11.43%). This made it difficult to discern any viable pattern. However, the knowledge of the diseases was mainly attributable to the breed type reared by farmer. Farmers raising Layer birds had a higher frequency of reporting Mareks disease, Fowl typhoid and IBD. These trends can be seen in the UpSet plots (Fig S6A). Litter disposal included dumping the litter either in pits, open area or drains lying in the vicinity of the farms. Based on UpSet plots, cleaning frequency of 30 days and disposal in pits is contributing to disease outbreaks. These patterns are also coinciding with the antimicrobial usage. All of the top patterns suggest the importance of biosecurity practices in managing outbreaks (Fig S6B).

## Discussion

Frequent disease outbreaks and logistic hurdles within the poultry sector have led to major economic losses in Pakistan. Farmers with little or no formal education were mainly involved in raising Broiler at a commercial level using controlled sheds which was associated with increased risk for disease outbreak. This is mainly because those who are associated with this profession are typically influential landowners in possession of larger areas with substantial capital to invest. Nonetheless, lack of education becomes a hindrance to disease control. On the contrary, educated farmers, which are considerably fewer, prefer open shed systems and have a mindset that rearing birds in an open environment leads to the best performing birds which reduces disease outbreaks. This corroborates with the previous study [25] which reveals that the education of farmers affects the technical efficiency of poultry farmers in Pakistan.

We found no association between lack of training and prevalence of disease, suggesting on-the-job learning. Previous studies have also demonstrated that high disease risk awareness does not necessarily translate to improved general farming practices [26, 27]. Furthermore, to cut losses, farmers tend to sell their poultry stock as quickly as possible. It has been reported that poultry producers prefer to market alive or depopulated birds in case of known/suspected infection to curb the disease but may result in further disease spread [28].

In open sheds, unrestricted movement of workers, unscheduled vaccination, and improper cleaning contribute to low biosecurity. In contrast, amongst semi-controlled and controlled sheds, movement of workers is restricted due to the automation of feeding and drinking systems. In addition, regular monitoring and record of diseased/dead birds along with proper vaccination schedule is maintained. A previous study from Bangladesh [29] showed substantial decrease in risk of AI outbreaks with the implementation of biosecurity practices. In another study [30], similar biosecurity practices in small scale poultry units also decreased disease risk. It should be noted that the prevalent biosecurity practices typically vary between the farms, and also differ when raising different poultry species [31].

We found that higher stocking density is a major risk factor for disease outbreaks. It has been reported that farmers house their birds in super intensive conditions to increase profit but the overcrowding ultimately increases birds susceptibility to infections and microbial attack [32, 33]. We highlight cleaning frequency and the litter disposal method to be major risk factors for disease spread (also corroborated by a previous survey conducted in Ethiopia and Switzerland [34, 35]). Furthermore, poor poultry waste management may cause health problems in flock, and contamination of land and water. Hence, appropriate farm waste disposal is crucial for protecting environment, human health and poultry welfare [31].

In majority of controlled sheds raising Broiler, where maximum disease outbreak has been reported, and rice husk is used as bedding material. Farmers using wood shaving as bedding material reported less outbreaks, which is supported by the previous findings about antimicrobial properties of wood shaving and improved performance [36, 37].

It is well established that poultry farming relies on antimicrobials usage to control diseases [38]. In this study, antimicrobials use frequency was highest amongst experienced farmers raising Broiler in large, controlled sheds. We observed high macrolide use amongst various farm types. Macrolides are broad spectrum antibiotics that are often used in chickens as therapeutic/prophylactic agents [39, 40]. The rampant use of antimicrobials in farms has resulted in the emergence of multiple antibiotic resistant bacterial strains from poultry sources [41]. We also reported aminoglycosides usage amongst poultry farmers. Aminoglycosides usage in veterinary medicine is associated with increased resistance in bacteria from clinical and animal origin [42, 43]. A similar increase in aminoglycoside resistance amongst bacterial strains of poultry origin from Pakistani farms is also observed [44, 45].

We also observed that farmers frequently supplement feeds with antimicrobials to enhance feed conversion rates. It has been suggested that antimicrobials help birds in gaining weight by various mechanisms such as immune system modulations and less energy uptake by gut bacteria but coincides with emergence of antimicrobial resistant bacteria and severe health challenges [46–48]. As a result, highest antimicrobial resistant zoonotic pathogen burden has been observed in low and middle income countries through poultry [49]. A few recently published studies accentuated high use of antimicrobials in livestock sector, specifically in commercial Broiler in Pakistan [9, 50]. The high antimicrobial usage in Pakistani poultry farms is due to the availability of antimicrobials without prescription, inaccurate diagnostic approaches, or lack of access to diagnostic facility [51, 52].

In the present survey, a high percentage of farmers reported ND, AI and FT outbreaks. Previously, several ND outbreaks have been reported in Pakistan, resulting in huge economic losses [53]. It has been established that the commercial poultry birds are highly susceptible to ND and it is endemic in six continents including Asia [54, 55]. High risk of AI outbreak has also been observed in large scale poultry farms compared to backyard flocks in Thailand [56]. In Pakistan, similar to ND, AI is also endemic and its high prevalence has been reported in various studies [5, 6, 57–59]. Fowl typhoid is caused by *Salmonella gallinarum* which is highly prevalent in Pakistan and other developing countries, leading to huge mortality and subsequent economic losses [60]. ND was found as the most prevalent poultry disease in our survey, mostly within Broiler farms, followed by AI and FT, a similar trend has been observed in previous studies [61, 62]. Despite the limited sample size, owing to inaccessibility of many major commercial farming setups, the study highlights major gaps in farm management practices associated with the antimicrobial usage and disease spread.

## Conclusions

The study highlights major gaps in farmer routine practices on farm, knowledge, and formal education. High disease incidence was associated with poor management practices employed by large farm setups and choice of breed which can be used to design effective intervention strategies to curtail spread of disease while optimizing production in the country. Farmers with higher education, however, have fewer outbreaks and are better able to control the use of antibiotics. The present data can be used as a reference by animal health authorities for surveillance and strategy implementation to optimize quality food production in Pakistan.

## Supplementary Information

Additional file 1.

Additional file 2.

Additional file 3.

## Data Availability

The relevant data is provided as supplementary files i.e., Additional file 2 and Additional file 3.
