# Supplementary material for: A cross-sectional survey of poultry management systems, practices and antimicrobial use in relation to disease outbreak in Pakistan
Source: BMC Res Notes. 2025 Apr 8;18:144. doi: 10.1186/s13104-025-07220-4 (PMC11977947; doi:10.1186/s13104-025-07220-4)
Supplement: Supplementary file 1 — Additional file 1. [file 13104_2025_7220_MOESM1_ESM.docx]

Supporting Information

**Fig. S1.** Significant dependences recovered between categorical-response questions in self-reported questionnaire using $\chi^{2}$ test of independence. Where significant (p<0.05), the $\chi^{2}$ Pearson residuals are calculated. The positive value is represented by the blue color, is a positive attraction between the corresponding row and column variables whilst negative values imply a repulsion (negative association; red) between the corresponding row and column variables.

**Fig. S2.** Continuation of $\chi^{2}$ test of independence results for significant dependence between questions in the self-reported questionnaire. See the legend of Supplementary Figure S1 for details.

**Fig. S3.** Continuation of $\chi^{2}$ test of independence results for significant dependence between questions in the self-reported questionnaire. See the legend of Supplementary Figure S1 for details.

**Fig. S4.** Continuation of $\chi^{2}$ test of independence results for significant dependence between questions in the self-reported questionnaire. See the legend of Supplementary Figure S1 for details.

**Table S1.** Major risk predictors associated with Avian influenza outbreak at poultry farms by fitting different regression models represented by M numbers. The significant predictors that cause an increase in disease outbreak are shown with red background, whilst those that cause a decrease in outbreak are shown with a blue background as compared to reference (REF), with the prevalence ratio/risk ratio shown in bold.

| **Predictor** | | ***β-* coefficients** | **Count (n=140)** | **Avian influenza outbreak, n=20 (14.29%)** | **Prevalence Ratio**  **(95% CI)** | **Significance** |
| --- | --- | --- | --- | --- | --- | --- |
| **M1** | Intercept | | NA |  | 0.28 (0.13 – 0.59) | *** |
|  | Farmer’s Education | Secondary | 18 | 5 (27.78%) | REF | NA |
|  |  | Higher secondary | 35 | 9 (25.71%) | 0.93 (0.36 – 2.36) | NS |
|  |  | Graduation | 56 | 4 (7.14%) | **0.26** (0.08 – 0.86) | * |
|  |  | Post-graduation | 31 | 2 (6.45%) | 0.23 (0.05 – 1.08) | NS |
| **M2** | Intercept | | NA |  | 0.03 (0.00– 0.23) | *** |
|  | Farming Experience | Less than 5 years | 30 | 1 (3.33%) | REF | NA |
|  |  | 5 to 10 years | 65 | 7 (10.77%) | 3.23 (0.42 – 25.10) | NS |
|  |  | More than 10 years | 45 | 12 (26.67%) | **8.00** (1.10 – 58.35) | * |
| **M3** | Intercept | | NA |  | 0.17 (0.10 – 0.29) | *** |
|  | Epidemic Training Status | Untrained | 66 | 11 (16.67%) | REF | NA |
|  |  | Self-research | 23 | 1 (4.35%) | 0.26 (0.04 – 1.91) | NS |
|  |  | Trained | 51 | 8 (15.69%) | 0.94 (0.41 – 2.17) | NS |
| **M4** | Intercept |  | NA |  | 0.27 (0.15 – 0.48) | *** |
|  | Farm Type | Controlled | 30 | 8 (26.67%) | REF | NA |
|  |  | Semi controlled | 48 | 9 (18.75%) | 0.70 (0.30 – 1.62) | NS |
|  |  | Open | 62 | 3 (4.84%) | **0.18** (0.05 – 0.64) | ** |
| **M5** | Intercept |  | NA |  | 0.20 (0.12 – 0.32) | *** |
|  | Breed Type | Broiler | 66 | 13 (19.70%) | REF | NA |
|  |  | Layer | 36 | 4 (11.11%) | 0.56 (0.20 – 1.60) | NS |
|  |  | Desi and crosses | 38 | 3 (7.90%) | 0.40 (0.12 – 1.32) | NS |
| **M6** | Intercept |  | NA |  | 0.11 (0.02 – 0.71) | * |
|  | Confinement Type | No confinement | 9 | 1 (11.11%) | REF | NA |
|  |  | Open house with mesh | 58 | 2 (3.45%) | 0.31 (0.03 – 3.08) | NS |
|  |  | Closed house with solid walls | 73 | 17 (23.29%) | 2.10 (0.32 – 13.93) | NS |
| **M7** | Intercept | | NA |  | 0.18 (0.11 – 0.29) | *** |
|  | Flock Management | All-in-all-out | 74 | 13 (17.57%) | REF | NA |
|  |  | Multiple flocks and all-in-all-out | 55 | 6 (10.91%) | 0.62 (0.25 – 1.53) | NS |
|  |  | Continuous topping | 11 | 1 (9.10%) | 0.52 (0.07 – 3.58) | NS |
| **M8** | Intercept | | NA |  | 0.08 (0.02 – 0.30) | *** |
|  | Cleaning Frequency | Every 5 days | 25 | 2 (8.00%) | REF | NA |
|  |  | Every 10 days | 48 | 5 (10.42%) | 1.30 (0.27 – 6.24) | NS |
|  |  | Every 30 days | 67 | 13 (19.40%) | **2.43** (0.59 – 9.99) | * |
| **M9** | Intercept | | NA |  | 0.50 (0.22 – 1.11) | NS |
|  | Litter Disposal | Open place | 6 | 3 (50.00%) | REF | NA |
|  |  | Drain | 36 | 5 (13.89%) | **0.28** (0.09 – 0.87) | * |
|  |  | Pit | 98 | 12 (12.24%) | **0.24** (0.09 – 0.64) | ** |
| **M10** | Intercept | | NA |  | 0.05 (0.02 – 0.14) | *** |
|  | Major Feed Ingredient: Canola | No | 73 | 4 (5.48%) | REF | NA |
|  |  | Yes | 67 | 16 (23.88%) | **4.36** (1.53 – 12.28) | ** |
| **M11** | Intercept |  | NA |  | 0.26 (0.16 – 0.42) | *** |
|  | Major Feed Ingredient: Wheat | No | 46 | 12 (26.09%) | REF | NA |
|  |  | Yes | 94 | 8 (8.51%) | **0.33** (0.14 – 0.74) | ** |
| **M12** | Intercept |  | NA |  | 0.75 (0.43 – 1.32) | NS |
|  | Major Feed Ingredient: Maize | No | 4 | 3 (75.00%) | REF | NA |
|  |  | Yes | 136 | 17 (12.50%) | **0.17** (0.08 – 0.34) | *** |
| **M13** | Intercept | | NA |  | 0.15 (0.08 – 0.29) | *** |
|  | Veterinary Clinic Access | No | 52 | 8 (15.38%) | REF | NA |
|  |  | Yes | 88 | 12 (13.64%) | 0.89 (0.39 – 2.03) | NS |
| **M14** | Intercept | | NA |  | 0.09 (0.05 – 0.17) | *** |
|  | Health Issue Staff | No | 109 | 10 (9.17%) | REF | NA |
|  |  | Yes | 31 | 10 (32.26%) | **3.52** (1.61 – 7.76) | ** |

**NA refers to Not Applicable; NS refers to Non-significant*

**p<0.05 **p<0.01 ***p<0.001*

**Table S2.** Major risk predictors associated with Newcastle disease outbreak at poultry farms by fitting different regression models represented by M numbers. The significant predictors that cause an increase in disease outbreak are shown with red background, whilst those that cause a decrease in outbreak are shown with a blue background as compared to reference (REF), with the prevalence ratio/risk ratio shown in bold.

| **Predictor** | | ***β-* coefficients** | **Count (n=140)** | **Newcastle disease outbreak, n=38 (27.14%)** | **Prevalence Ratio**  **(95% CI)** | **Significance** |
| --- | --- | --- | --- | --- | --- | --- |
| **M1** | Intercept |  | NA |  | 0.39 (0.22 – 0.69) | ** |
|  | Farmer’s Education | Secondary | 18 | 7 (38.89%) | REF | NA |
|  |  | Higher secondary | 35 | 12 (34.29%) | 0.88 (0.42 – 1.85) | NS |
|  |  | Graduation | 56 | 15 (26.79%) | 0.69 (0.33 – 1.42) | NS |
|  |  | Post-graduation | 31 | 4 (12.90%) | **0.33** (0.11 – 0.98) | * |
| **M2** | Intercept | | NA |  | 0.17 (0.07– 0.37) | *** |
|  | Farming Experience | Less than 5 years | 30 | 5 (16.67%) | REF | NA |
|  |  | 5 to 10 years | 65 | 14 (21.54%) | 1.29 (0.51 – 3.26) | NS |
|  |  | More than 10 years | 45 | 19 (42.22%) | **2.53** (1.06 – 6.05) | * |
| **M3** | Intercept |  | NA |  | 0.26 (0.17 – 0.39) | *** |
|  | Epidemic Training Status | Untrained | 66 | 17 (25.76%) | REF | NA |
|  |  | Self-research | 23 | 9 (39.13%) | 1.52 (0.79 – 2.92 ) | NS |
|  |  | Trained | 51 | 12 (23.53%) | 0.91 (0.48 – 1.74) | NS |
| **M4** | Intercept |  | NA |  | 0.50 (0.35 – 0.72) | *** |
|  | Farm Type | Controlled | 30 | 15 (50.00%) | REF | NA |
|  |  | Semi controlled | 48 | 11 (22.92%) | **0.46** (0.24 – 0.86) | * |
|  |  | Open | 62 | 12 (19.36%) | **0.39** (0.21 – 0.72) | ** |
| **M5** | Intercept |  | NA |  | 0.26 (0.16 – 0.42) | *** |
|  | Labor Capacity | Less than 3 | 50 | 13 (26.00%) | REF | NA |
|  |  | 3 to 5 | 57 | 10 (17.54%) | 0.67 (0.32 – 1.40) | NS |
|  |  | More than5 | 33 | 15 (45.45%) | 1.75 (0.96 – 3.18) | NS |
| **M6** | Intercept |  | NA |  | 0.39 (0.29 – 0.53) | *** |
|  | Breed Type | Broiler | 66 | 26 (39.39%) | REF | NA |
|  |  | Layer | 36 | 3 (8.33%) | **0.21** (0.07 – 0.65) | ** |
|  |  | Desi and crosses | 38 | 9 (23.68%) | 0.60 (0.32 – 1.15) | NS |
| **M7** | Intercept | | NA |  | 0.11 (0.02 – 0.71) | * |
|  | Confinement Type | No confinement | 9 | 1 (11.11%) | REF | NA |
|  |  | Open house with mesh | 58 | 13 (22.41%) | 2.02 (0.30 – 13.61) | NS |
|  |  | Closed house with solid walls | 73 | 24 (32.88%) | 2.96 (0.45 – 19.33) | NS |
| **M8** | Intercept | | NA |  | 0.36 (0.27 – 0.49) | *** |
|  | Flock Management | All-in-all-out | 74 | 27 (36.49%) | REF | NA |
|  |  | Multiple flocks and all-in-all-out | 55 | 8 (14.55%) | **0.40** (0.20 – 0.81) | * |
|  |  | Continuous topping | 11 | 3 (3.00%) | 0.75 (0.27 – 2.05) | NS |
| **M9** | Intercept | | NA |  | 0.20 (0.09 – 0.44) | *** |
|  | Cleaning Frequency | Every 5 days | 25 | 5 (20.00%) | REF | NA |
|  |  | Every 10 days | 48 | 8 (16.67%) | 0.83 (0.30 – 2.28) | NS |
|  |  | Every 30 days | 67 | 25 (37.31%) | 1.87 (0.80 – 4.34) | NS |
| **M10** | Intercept | | NA |  | 0.16 (0.10 – 0.28) | *** |
|  | Major Feed Ingredient: Canola | No | 73 | 12 (16.44%) | REF | NA |
|  |  | Yes | 67 | 26 (38.81%) | 2.36 (1.30 – 4.29) | ** |
| **M11** | Intercept | | NA |  | 0.30 (0.20 – 0.47) | *** |
|  | Major Feed Ingredient: Wheat | No | 46 | 14 (30.43%) | REF | NA |
|  |  | Yes | 94 | 24 (25.53%) | 0.84 (0.48 – 1.46) | NS |
| **M12** | Intercept | | NA |  | 0.25 (0.05 – 1.36) | NS |
|  | Major Feed Ingredient: Maize | No | 4 | 1 (25.00%) | REF | NA |
|  |  | Yes | 136 | 37 (27.21%) | 1.09 (0.19 – 6.07) | NS |
| **M13** | Intercept | | NA |  | 0.21 (0.13 – 0.36) | *** |
|  | Veterinary Clinic Access | No | 52 | 11 (21.15%) | REF | NA |
|  |  | Yes | 88 | 27 (30.68%) | 1.45 (0.79 – 2.67) | NS |
| **M14** | Intercept | | NA |  | 0.22 (0.15 – 0.31) | *** |
|  | Health Issue Staff | No | 109 | 24 (22.02%) | REF | NA |
|  |  | Yes | 31 | 14 (45.16%) | **2.05** (1.21 – 3.47) | ** |

**NA refers to Not Applicable; NS refers to Non-significant*

**p<0.05 **p<0.01 ***p<0.001*

**Table S3.** Major risk predictors associated with Fowl typhoid outbreak at poultry farms by fitting different regression models represented by M numbers. The significant predictors that cause an increase in disease outbreak are shown with red background, whilst those that cause a decrease in outbreak are shown with a blue background as compared to reference (REF), with the prevalence ratio/risk ratio shown in bold.

| **Predictor** | | ***β-* coefficients** | **Count (n=140)** | **Fowl typhoid outbreak, n=18 (12.86%)** | **Prevalence Ratio**  **(95% CI)** | **Significance** |
| --- | --- | --- | --- | --- | --- | --- |
| **M1** | Intercept | | NA |  | 0.06 (0.01 – 0.37) | ** |
|  | Farmer’s Education | Secondary | 18 | 1 (5.56%) | REF | NA |
|  |  | Higher secondary | 35 | 7 (20.00%) | 3.60 (0.48 – 27.05) | NS |
|  |  | Graduation | 56 | 8 (14.29%) | 2.57 (0.34 – 19.19) | NS |
|  |  | Post-graduation | 31 | 2 (6.45%) | 1.16 (0.11– 11.93) | NS |
| **M2** | Intercept | | NA |  | 0.07 (0.02– 0.25) | *** |
|  | Farming Experience | Less than 5 years | 30 | 2 (6.67%) | REF | NA |
|  |  | 5 to 10 years | 65 | 6 (9.23%) | 1.38 (0.30 – 6.46) | NS |
|  |  | More than 10 years | 45 | 10 (22.22%) | 3.33 (0.78 – 14.16) | NS |
| **M3** | Intercept | | NA |  | 0.14 (0.07 – 0.25) | *** |
|  | Epidemic Training Status | Untrained | 66 | 9 (13.63%) | REF | NA |
|  |  | Self-research | 23 | 3 (13.04%) | 0.96 (0.28 – 3.23) | NS |
|  |  | Trained | 51 | 6 (11.76%) | 0.86 (0.33 – 2.27 | NS |
| **M4** | Intercept | | NA |  | 0.17 (0.07 – 0.37) | *** |
|  | Farm Type | Controlled | 30 | 5 (16.67%) | REF | NA |
|  |  | Semi controlled | 48 | 5 (10.41%) | 0.63 (0.20 – 1.98) | NS |
|  |  | Open | 62 | 8 (12.90%) | 0.77 (0.28 – 2.17) | NS |
| **M5** | Intercept | | NA |  | 0.14 (0.07 – 0.25) | *** |
|  | Breed Type | Broiler | 66 | 9 (13.64%) | REF | NA |
|  |  | Layer | 36 | 8 (22.22%) | 1.63 (0.69 – 3.86) | NS |
|  |  | Desi and crosses | 38 | 1 (2.63%) | 0.19 (0.03 – 1.47) | NS |
| **M6** | Intercept | | NA |  | 0.11 (0.02 – 0.71) | * |
|  | Confinement Type | No confinement | 9 | 1 (11.11%) | REF | NA |
|  |  | Open house with mesh | 58 | 7 (12.07%) | 1.09 (0.15 – 7.82) | NS |
|  |  | Closed house with solid walls | 73 | 10 (13.70%) | 1.23 (0.18 – 8.54) | NS |
| **M7** | Intercept |  | NA |  | 0.14 (0.08 – 0.24) | *** |
|  | Flock Management | All-in-all-out | 74 | 10 (13.51%) | REF | NA |
|  |  | Multiple flocks and all-in-all-out | 55 | 7 (12.73%) | 0.94 (0.38 – 2.32) | NS |
|  |  | Continuous topping | 11 | 1 (9.09%) | 0.67 (0.10 – 4.76) | NS |
| **M8** | Intercept |  | NA |  | 0.08 (0.02 – 0.30) | *** |
|  | Cleaning Frequency | Every 5 days | 25 | 2 (8.00%) | REF | NA |
|  |  | Every 10 days | 48 | 7 (14.59) | 1.82 (0.41 – 8.13) | NS |
|  |  | Every 30 days | 67 | 9 (13.43%) | 1.68 (0.39 – 7.24) | NS |
| **M9** | Intercept |  | NA |  | 0.17 (0.03 – 1.00) | * |
|  | Litter Disposal | Open place | 6 | 1 (16.67%) | REF | NA |
|  |  | Drain | 36 | 5 (13.89%) | 0.83 (0.12 – 5.95) | NS |
|  |  | Pit | 98 | 12 (12.24%) | 0.73 (0.11 – 4.75) | NS |
| **M10** | Intercept |  | NA |  | 0.21 (0.13 – 0.33) | *** |
|  | Stocking Density | Less than 2 ft^2^/bird | 72 | 15 (20.83%) | REF | NA |
|  |  | 2 to 4 ft^2^/bird | 66 | 2 (3.03%) | **0.15** (0.03 – 0.61) | ** |
|  |  | More than 4 ft^2^/bird | 2 | 1 (50.00%) | 2.40 (0.56 – 10.31) | NS |
| **M11** | Intercept | | NA |  | 0.12 (0.07 – 0.23) | *** |
|  | Major Feed Ingredient: Canola | No | 73 | 9 (12.33%) | REF | NA |
|  |  | Yes | 67 | 9 (13.43%) | 1.09 (0.46 – 2.58) | NS |
| **M12** | Intercept |  | NA |  | 0.11 (0.05 – 0.25) | *** |
|  | Major Feed Ingredient: Wheat | No | 46 | 5 (10.87%) | REF | NA |
|  |  | Yes | 94 | 13 (13.83%) | 1.27 (0.48 – 3.35) | NS |
| **M13** | Intercept | | NA |  | 0.17 (0.10 – 0.31) | *** |
|  | Veterinary Clinic Access | No | 52 | 9 (17.31%) | REF | NA |
|  |  | Yes | 88 | 9 (10.23%) | 0.59 (0.25 – 1.39) | NS |
| **M14** | Intercept |  | NA |  | 0.11 (0.06 – 0.19) | *** |
|  | Health Issue Staff | No | 109 | 12 (11.01%) | REF | NA |
|  |  | Yes | 31 | 6 (19.36%) | 1.76 (0.72 – 4.30) | NS |

**NA refers to Not Applicable; NS refers to Non-significant*

**p<0.05 **p<0.01 ***p<0.00*

**Fig. S5.** Sample questionnaire used in the survey.

**Fig. S6.** UpSet plot indicating frequency-based sorted intersecting patterns of (A) Disease knowledge of the farmer and the breed raised (B) Biosecurity practices, disease outbreak, and antibiotic usage. The data is Boolean (Yes/No) with lines connecting those categories where the answers were given as “Yes”.

**Fig. S7.** UpSet plot indicating frequency-based sorted intersecting patterns of of (A) Farm setup and selective antibiotic usage (B) Farming experience and disease outbreak. The data is Boolean (Yes/No) with lines connecting those categories where the answers were given as “Yes”.
