# Supplementary material for: A cross-sectional survey of poultry management systems, practices and antimicrobial use in relation to disease outbreak in Pakistan
Source: BMC Res Notes. 2025 Apr 8;18:144. doi: 10.1186/s13104-025-07220-4 (PMC11977947; doi:10.1186/s13104-025-07220-4)
Supplement: Supplementary file 3 — Additional file 3. [file 13104_2025_7220_MOESM3_ESM.zip › Logbin_prevalence_ratio_data/Disease_Outbreak_ND/Health_Issue_Staff.html]

|  | Disease\_Outbreak\_ND | | | | | | |
| --- | --- | --- | --- | --- | --- | --- | --- |
| Predictors | Risk Ratios | std. Error | std. Beta | standardized std. Error | CI | standardized CI | Statistic |
| (Intercept) | 0.22 \*\*\* | 0.04 | 0.22 | 0.04 | 0.15 – 0.31 | 0.15 – 0.31 | -8.40 |
| Health Issue Staff [Yes] | 2.05 \*\* | 0.55 | 2.05 | 0.55 | 1.21 – 3.47 | 1.21 – 3.47 | 2.68 |
| Observations | 140 | | | | | | |
| R2 Nagelkerke | 0.062 | | | | | | |
| \* p<0.05   \*\* p<0.01   \*\*\* p<0.001 | | | | | | | |
