# Supplementary material for: A cross-sectional survey of poultry management systems, practices and antimicrobial use in relation to disease outbreak in Pakistan
Source: BMC Res Notes. 2025 Apr 8;18:144. doi: 10.1186/s13104-025-07220-4 (PMC11977947; doi:10.1186/s13104-025-07220-4)
Supplement: Supplementary file 3 — Additional file 3. [file 13104_2025_7220_MOESM3_ESM.zip › Logbin_prevalence_ratio_data/Health_Issue_Staff/Breed_Type.html]

|  | Health\_Issue\_Staff | | | | | | |
| --- | --- | --- | --- | --- | --- | --- | --- |
| Predictors | Risk Ratios | std. Error | std. Beta | standardized std. Error | CI | standardized CI | Statistic |
| (Intercept) | 0.29 \*\*\* | 0.06 | 0.29 | 0.06 | 0.20 – 0.42 | 0.20 – 0.42 | -6.43 |
| Breed Type [D] | 0.37 \* | 0.19 | 0.37 | 0.19 | 0.13 – 1.00 | 0.13 – 1.00 | -1.97 |
| Breed Type [L] | 0.77 | 0.28 | 0.77 | 0.28 | 0.38 – 1.58 | 0.38 – 1.58 | -0.71 |
| Observations | 140 | | | | | | |
| R2 Nagelkerke | 0.055 | | | | | | |
| \* p<0.05   \*\* p<0.01   \*\*\* p<0.001 | | | | | | | |
