# Supplementary material for: A cross-sectional survey of poultry management systems, practices and antimicrobial use in relation to disease outbreak in Pakistan
Source: BMC Res Notes. 2025 Apr 8;18:144. doi: 10.1186/s13104-025-07220-4 (PMC11977947; doi:10.1186/s13104-025-07220-4)
Supplement: Supplementary file 3 — Additional file 3. [file 13104_2025_7220_MOESM3_ESM.zip › Logbin_prevalence_ratio_data/Health_Issue_Staff/Farming_Experience.html]

|  | Health\_Issue\_Staff | | | | | | |
| --- | --- | --- | --- | --- | --- | --- | --- |
| Predictors | Risk Ratios | std. Error | std. Beta | standardized std. Error | CI | standardized CI | Statistic |
| (Intercept) | 0.07 \*\*\* | 0.05 | 0.07 | 0.05 | 0.02 – 0.25 | 0.02 – 0.25 | -3.96 |
| Farming Experience [F5T10] | 3.23 | 2.34 | 3.23 | 2.34 | 0.78 – 13.33 | 0.78 – 13.33 | 1.62 |
| Farming Experience [M10] | 5.00 \* | 3.57 | 5.00 | 3.57 | 1.23 – 20.30 | 1.23 – 20.30 | 2.25 |
| Observations | 140 | | | | | | |
| R2 Nagelkerke | 0.088 | | | | | | |
| \* p<0.05   \*\* p<0.01   \*\*\* p<0.001 | | | | | | | |
