# Supplementary material for: A cross-sectional survey of poultry management systems, practices and antimicrobial use in relation to disease outbreak in Pakistan
Source: BMC Res Notes. 2025 Apr 8;18:144. doi: 10.1186/s13104-025-07220-4 (PMC11977947; doi:10.1186/s13104-025-07220-4)
Supplement: Supplementary file 3 — Additional file 3. [file 13104_2025_7220_MOESM3_ESM.zip › Logbin_prevalence_ratio_data/Disease_Outbreak_FT/Education.html]

|  | Disease\_Outbreak\_FT | | | | | | |
| --- | --- | --- | --- | --- | --- | --- | --- |
| Predictors | Risk Ratios | std. Error | std. Beta | standardized std. Error | CI | standardized CI | Statistic |
| (Intercept) | 0.06 \*\* | 0.05 | 0.06 | 0.05 | 0.01 – 0.37 | 0.01 – 0.37 | -2.97 |
| Education [G] | 2.57 | 2.64 | 2.57 | 2.64 | 0.34 – 19.19 | 0.34 – 19.19 | 0.92 |
| Education [HS] | 3.60 | 3.70 | 3.60 | 3.70 | 0.48 – 27.05 | 0.48 – 27.05 | 1.24 |
| Education [PG] | 1.16 | 1.38 | 1.16 | 1.38 | 0.11 – 11.93 | 0.11 – 11.93 | 0.13 |
| Observations | 140 | | | | | | |
| R2 Nagelkerke | 0.051 | | | | | | |
| \* p<0.05   \*\* p<0.01   \*\*\* p<0.001 | | | | | | | |
