# Supplementary material for: A cross-sectional survey of poultry management systems, practices and antimicrobial use in relation to disease outbreak in Pakistan
Source: BMC Res Notes. 2025 Apr 8;18:144. doi: 10.1186/s13104-025-07220-4 (PMC11977947; doi:10.1186/s13104-025-07220-4)
Supplement: Supplementary file 3 — Additional file 3. [file 13104_2025_7220_MOESM3_ESM.zip › Logbin_prevalence_ratio_data/Disease_Outbreak/Knowledge_Fowl_Typhoid.html]

|  | Disease\_Outbreak | | | | | | |
| --- | --- | --- | --- | --- | --- | --- | --- |
| Predictors | Risk Ratios | std. Error | std. Beta | standardized std. Error | CI | standardized CI | Statistic |
| (Intercept) | 0.50 \*\*\* | 0.07 | 0.50 | 0.07 | 0.39 – 0.65 | 0.39 – 0.65 | -5.28 |
| Knowledge Fowl Typhoid [Yes] | 1.15 | 0.19 | 1.15 | 0.19 | 0.83 – 1.58 | 0.83 – 1.58 | 0.84 |
| Observations | 140 | | | | | | |
| R2 Nagelkerke | 0.007 | | | | | | |
| \* p<0.05   \*\* p<0.01   \*\*\* p<0.001 | | | | | | | |
