# Supplementary material for: A cross-sectional survey of poultry management systems, practices and antimicrobial use in relation to disease outbreak in Pakistan
Source: BMC Res Notes. 2025 Apr 8;18:144. doi: 10.1186/s13104-025-07220-4 (PMC11977947; doi:10.1186/s13104-025-07220-4)
Supplement: Supplementary file 3 — Additional file 3. [file 13104_2025_7220_MOESM3_ESM.zip › Logbin_prevalence_ratio_data/Disease_Outbreak/Stocking_Density.html]

|  | Disease\_Outbreak | | | | | | |
| --- | --- | --- | --- | --- | --- | --- | --- |
| Predictors | Risk Ratios | std. Error | std. Beta | standardized std. Error | CI | standardized CI | Statistic |
| (Intercept) | 0.72 \*\*\* | 0.05 | 0.72 | 0.05 | 0.63 – 0.83 | 0.63 – 0.83 | -4.45 |
| Stocking Density [F2T4] | 0.48 \*\*\* | 0.09 | 0.48 | 0.09 | 0.34 – 0.69 | 0.34 – 0.69 | -3.97 |
| Stocking Density [M4] | 0.69 | 0.49 | 0.69 | 0.49 | 0.17 – 2.79 | 0.17 – 2.79 | -0.52 |
| Observations | 140 | | | | | | |
| R2 Nagelkerke | 0.177 | | | | | | |
| \* p<0.05   \*\* p<0.01   \*\*\* p<0.001 | | | | | | | |
